# The acute effects of thermogenic fitness drink formulas containing 140 mg and 100 mg of caffeine on energy expenditure and fat metabolism at rest and during exercise

**DOI:** 10.1186/s12970-020-0341-4

**Published:** 2020-02-13

**Authors:** Nicolas W. Clark, Adam J. Wells, Nicholas A. Coker, Erica R. Goldstein, Chad H. Herring, Tristan M. Starling-Smith, Alyssa N. Varanoske, Valeria L. G. Panissa, Jeffrey R. Stout, David H. Fukuda

**Affiliations:** 1 School of Kinesiology and Physical Therapy, Institute of Exercise Physiology and Rehabilitation Science, University of Central Florida, Orlando, Florida USA; 2 Department of Sport, School of Physical Education and Sport, University of São Paulo, São Paulo, Brazil

**Keywords:** Caffeine, Energy drinks, Caloric expenditure, Fat oxidation, Exercise

## Abstract

**Background:**

Thermogenic fitness drink formulas (TFD) have been shown to increase energy expenditure and markers of lipid metabolism. The purpose of the current study was to compare TFD formulas containing different caffeine concentrations versus a placebo drink on energy expenditure and lipid metabolism at rest and during exercise.

**Methods:**

Thirty-two recreationally active participants (22.9 ± 0.7 y, 167.1 ± 1.4 cm, 68.8 ± 2.0 kg, 24.0 ± 1.2% fat) who were regular caffeine consumers, participated in this randomized, double-blind, crossover design study. Participants reported to the laboratory on three occasions, each of which required consumption of either a TFD containing 140 mg or 100 mg of caffeine or a placebo. Baseline measurements of resting energy expenditure (REE) and resting fat oxidation (RFO) were assessed using indirect calorimetry as well as measurements of serum glycerol concentration. Measurements were repeated at 30, 60, 90 min post-ingestion. Following resting measures, participants completed a graded exercise test to determine maximal oxygen uptake (V̇O_2max_), maximal fat oxidation (MFO) and the exercise intensity that elicits MFO (Fat_max_), and total energy expenditure (EE).

**Results:**

A significant interaction was shown for REE (*p* < 0.01) and RFO (*p* < 0.01). Area under the curve analysis showed an increased REE for the 140 mg compared to the 100 mg formula (*p* = 0.02) and placebo (*p* < 0.01) and an increased REE for the 100 mg formula compared to placebo (*p* = 0.02). RFO significantly decreased for caffeinated formulas at 30 min post ingestion compared to placebo and baseline (*p* < 0.01) and significantly increased for the 140 mg formula at 60 min post-ingestion (*p* = 0.03). A main effect was shown for serum glycerol concentrations over time (*p* < 0.01). No significant differences were shown for V̇O_2max_ (*p* = 0.12), Fat_max_ (*p* = 0.22), and MFO (*p* = 0.05), and EE (*p* = 0.08) across drinks.

**Conclusions:**

Our results suggest that TFD formulas containing 100 and 140 mg of caffeine are effective in increasing REE and that a 40 mg of caffeine difference between the tested formulas may impact REE and RFO in healthy individuals within 60 min of ingestion.

## Background

Caffeine (1, 3, 7-trimethylxanthine) is a natural substance occurring in the seeds, leaves, and fruits of over 60 plants and is the most widely consumed psychostimulant in the world [1–3]. According to Fulgoni et al. [1], nearly 89% of the adult population in the United States consumes caffeine in the form of food, beverages, medication, and dietary supplements. Among caffeinated beverages, energy drinks are estimated to represent a small share of 3–10% of all age consumers [4–8]. The energy drink market, however, has grown 240% between the years of 2004–2009, which makes it one of the fastest growing nutrition markets in the United States [3, 6, 8–10].

Energy drinks may contain caffeine from a wide variety of sources, in addition to other bioactive ingredients (e.g., catechin polyphenols) that are purportedly added to increase physical stamina and promote mental alertness [11]. Among some of the different formulations of energy drinks, thermogenic fitness drinks (TFD) typically contain blends of caffeine-containing substances such as green tea and guarana extracts that are marketed with the intent to support weight loss. For example, a blend of these components has been shown to be effective in increasing daily energy expenditure by 8% when consumed before a meal three times per day, as compared to placebo [12]. Caffeine is a stimulant of the central nervous system [13]. Sympathoadrenal system activation resulting in increased epinephrine concentration, has the potential to increase lipid mobilization and consequently lipolysis [14]. Previous research with TFD containing 200 mg of caffeine have shown increased resting energy expenditure, circulating glycerol, and free fatty acids [15]. In addition to an increased thermogenic effect during rest [16], caffeine has been shown to potentially increase the rate of fat oxidation, while separately enhancing exercise performance [17, 18]; although, less is known about the metabolic response to a TFD during exercise [19].

The amount of caffeine occurring naturally in both coffee and tea is highly variable (e.g. roast, product, tea leaf, etc.). Energy drinks, energy shots, and—under the same category—TFDs contain lower variability in the amount of caffeine as part of the formulas (approximately 15%) [20]. Recently, Benson et al. [4] reported that the overall national average for caffeine consumption was 195 mg/day, which is above the 50th percentile (143 mg/day) reported in the NHANES 2013–2016 data. Caffeine may have anxiogenic effects in some individuals and previous studies have shown that overall consumption is moderated by caffeine concentration per drink, with fewer drinks consumed with greater amounts of caffeine per beverage [5, 21, 22]. A concern exists within the literature regarding risk for severe medical events with caffeine consumption above 400 mg, which is the Food and Drug Administration’s (FDA) maximum recommended daily amount of caffeine [23, 24].

Studies that have examined caffeine consumption on an hourly basis have demonstrated that beverages with higher caffeine concentrations, such as coffee, are disproportionately consumed in the morning with relatively less caffeinated products ingested throughout the remainder of the day [4, 25]. It is plausible that the availability of lower absolute doses of caffeine in TFD could be useful in allowing consumers to selectively moderate both intake and timing, while lowering risk for adverse side effects due to unintentional excessive consumption, especially if a minimal threshold (or range of caffeine) could be identified that produces thermogenic and ergogenic effects like highly caffeinated products.

The present study examined the acute effects of two different commercially available TFD formulas containing 140 mg and 100 mg and a placebo drink on metabolism during rest and exercise. We hypothesized that both caffeinated formulas would display an increase in energy expenditure as measured by indirect calorimetry and serum concentration of glycerol at rest compared to placebo, but no differences would be noted among caffeinated formulas. We also hypothesized that caffeinated formulas would increase maximal oxygen uptake, maximal fat oxidation and the exercise intensity that elicits maximal fat oxidation, and energy expenditure (EE) across drinks when compared to a placebo, as measured by indirect calorimetry, during a graded exercise test.

## Methods

### Experimental protocol

**Fig. 1:**
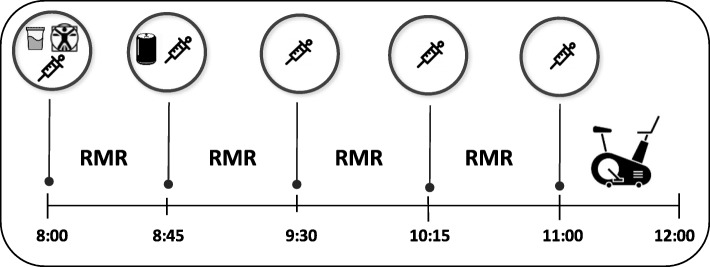
Experimental design of the study; = hydration test,  = anthropometrics and body composition,  = blood draw, RMR= resting metabolic rate,  = thermogenic fitness drink formula,  =graded exercise test

Three testing visits separated by a minimum of 48 h were completed within a two-week period. The timeline for each testing visit is outlined in Fig. 1. Participants were asked to maintain a consistent diet and track their food and beverage intake for the entire day prior to each of the three testing visits. Each testing visit was scheduled in the morning between 8 am, and 9 am, following an 8-h fast with no caffeine consumption and a 24-h period of no exercise or alcohol consumption. Baseline hydration status was evaluated upon arrival to the laboratory. After assessing height, body mass, and body composition, participants were led to a calm and quiet environment for baseline measurements consisting of a baseline blood draw for determining serum glycerol concentration followed by analysis of resting metabolic rate. A randomized, double-blind, crossover design was employed where participants were assigned to complete three trials, each of which required consumption of one of the following beverages:

- 140 mg formula (10 kcal drink containing a total of 140 mg of caffeine from a proprietary blend of caffeine, guarana, ginger, and green tea extract containing EGCG),
- 100 mg formula (10 kcal drink containing a total of 100 mg of caffeine from a proprietary blend of caffeine, guarana, ginger, and green tea extract containing EGCG),
- Placebo (artificially sweetened non-caloric/non-caffeinated drink).

Assessments were repeated at 30, 60, and 90 min following consumption of each beverage. Immediately following the last resting measurements, a graded exercise test was conducted to determine metabolic responses and performance outcomes.

### Participants

Thirty-two recreationally active men (*n* = 15) and women (*n* = 17) between the ages of 18 and 35 years old who were regular caffeine consumers of no more than 250 mg per day were recruited to participate in this research investigation (Table 1). After participants signed the informed consent they completed the Physical Activity Readiness Questionnaire (PARQ+), medical and activity history questionnaire (MHQ), and a caffeine consumption questionnaire adapted from Landrum [26]. This study was approved by the university’s Institutional Review Board. Participants were excluded if they had any physical limitations, metabolic diseases, were caffeine naïve or consumed more than 250 mg of caffeine per day according to the caffeine consumption questionnaire, and/or did not meet the ACSM recommendation of at least 150 min of exercise per week for the past 6 months [27].

**Table 1:** Participant demographics

Age (years)	22.9 ± 0.7
Height (centimeters)	167.1 ± 1.4
Weight (kilograms)^a^	68.8 ± 2.0
Body fat (%)^a^	24.0 ± 1.2
Total energy intake (kcal)^a^	1918 ± 127.6
Caffeine intake (g/week)	832 ± 69

Data [(n = 32 except for Total Energy Intake (*n* = 27)] are expressed as means ± SE

^a^average across testing visits

### Nutrient intake and dietary recall

Participants were required to complete a 24-h dietary recall. Dietary intake data for 24-h recalls were collected and analyzed using the Automated Self-Administered 24-h (ASA24) dietary assessment tool (version 2018, National Cancer Institute, Bethesda, MD) [28]. The ASA24 dietary recall assessment was utilized to estimate mean total energy intake (TEI) in kilocalories (Kcal) before each testing day. Participants were provided with a login and a password and detailed tutorial on how to access and complete the ASA24. The dietary recall was completed the night before each testing visit and after the last food item or drink consumed. During the recall, participants received automated prompts that would assist them in quantifying portion sizes, actual volume of food consumed at each meal or snack, and commonly forgotten items (condiments, supplements, sugar-sweetened beverages). A total of 27 participants complied with the dietary recall instructions and were included in the data analysis.

### Hydration status, anthropometrics, and body composition

Participants were asked to refrain from food or drink consumption—except water—for 8 h prior to testing and to be euhydrated. Urine samples were analyzed for hydration status using the refractometry method (Human Urine Refractometer, MISCO Refractometer, Cleveland, OH, USA). Participants could not initiate testing until proper hydration was confirmed, and specific gravity of the urine was less than or equal to 1.020. Following hydration testing, height was assessed using a stadiometer (500KL Health O Meter, Alsip, IL, USA). Body fat percentage (%BF) was estimated using a multi-frequency bioelectrical impedance analysis device (InBody 770, InBody, Seoul, Korea) and body mass (BM) was measured with a built-in scale. Participants were tested wearing minimal clothing and barefoot without socks.

### Resting metabolic rate testing

Resting metabolic rate (RMR) was measured using an automated metabolic gas analysis system (TrueOne 2400, Parvo Medics, Sandy, Utah, USA) to examine changes in whole-body metabolism after drink ingestion. After hydration status and body composition measurements were obtained, participants were led to a calm, quiet, mild-light, temperature (21–24 °C) controlled environment. Participants were instructed to lie in a supine position while enclosed in a clear hard plastic canopy, which was attached to the metabolic cart and dilution pump via a breathing tube. Oxygen uptake (V̇O_2_) and carbon dioxide production (V̇CO_2_) were measured for 30 min at baseline and for 20 min at the 30-, 60-, and 90-min time points post-ingestion. Respiratory gas values were averaged over one-minute intervals and posteriorly averaged for the last 10 min of each time point to estimate resting energy expenditure (REE). Total REE was also estimated by conducting area under the curve analyses over the 90-min procedure. As recommended by the manufacturer, a non-protein stoichiometric equation was used to estimate resting fat oxidation rate (RFO) (1.695 · V̇O_2_–1.701 · V̇CO_2_) [29].

### Blood venous sampling and glycerol analysis

Venous blood was obtained during rest from the antecubital area of the arm using a Teflon cannula with a three-way stopcock with a male luer lock adapter. The cannula was maintained patent using a non-heparinized isotonic saline solution for the duration of the trial. A total of four blood draws occurred for each trial (baseline, 30, 60, and 90 min post-ingestion) collected in two 10 mL serum Vacutainer® tubes. Following a given blood draw, the tube was allowed to clot for 30 min followed by centrifugation at 4000 x g for 15 min. Serum samples were placed into separate 1.8-mL microcentrifuge tubes and stored at -80°C in the Exercise Biochemistry Lab for later analysis. Serum glycerol was determined via direct enzymatic analysis using a commercially available assay (Clinical Glycerol II Reagent Kit GMRD-177; Analox Instruments Ltd., Stourbridge, UK). All samples for each assay were thawed once and analyzed in duplicate by the same technician to reduce potential inter-assay variance (CV:7.3%). Due to technical issues, glycerol concentration analyses were not completed for three participants.

### Graded exercise test, indirect calorimetry, and calculations

Participants performed a graded exercise test to exhaustion (GXT) on an electromagnetically-braked cycle ergometer (Corival, Lode B.V., Groningen, Netherlands). The GXT protocol consisted of a 10-min warm-up at 50 watts for male participants and 30 watts for female participants. Work rate was increased by 35 watts for males and 25 watts for females every 3 min until volitional fatigue. Breath-by-breath gas exchange data were collected using a metabolic gas analyzer (K-5 CPET, Cosmed, Rome, Italy) and used to determine maximal oxygen uptake (V̇O_2max_) and total energy expenditure during exercise (EE). The rating of perceived exertion from Borg’s 10-point scale was recorded during each stage of the GXT and immediately upon completion to confirm maximal exertion [30]. Average values for V̇O_2_ and V̇CO_2_ for the last minute of each stage were calculated using stoichiometric equations and used to determine fat oxidation, while assuming negligible protein oxidation [31]. Maximal fat oxidation (MFO) and the exercise intensity at which MFO occurred (Fat_max_) were then determined using a third order polynomial function for each participant [32]. Two participants did not complete the GXT due to technical issues and Fat_max_ could not be obtained for an additional two participants; therefore, a total of 28 participants were included in the final analysis.

### Statistical analysis

All analyses were conducted with an open-source statistical analysis software program (JASP; version 0.9). Alpha level was set a priori at *p* < 0.05. Data were assessed for sphericity and in case the assumption was violated, Greenhouse-Geisser correction was applied. Total energy intake, BM, %BF, REE, as well as V̇O_2 max_, EE, Fat_max_, and MFO were compared using separate one-way repeated analysis of variance (ANOVA). Resting energy expenditure, RFO, and blood glycerol were evaluated using a two-way (trial × time) repeated measures analysis of variance. If a significant difference (*p* < 0.05) was observed, Holm post hoc analyses were conducted, and effect sizes were calculated as Cohen’s d values. Follow-up one-way repeated measure ANOVAs were used to reveal differences across trials and time points when necessary.

## Results

### Nutritional intake, anthropometrics, and body composition

No significant differences were found for TEI (*p* = 0.27), BM (*p* = 0.77), and %BF (*p* = 0.32) across visits. Participant demographics are provided in Table 1.

### Resting metabolic rate

**Fig. 2:**
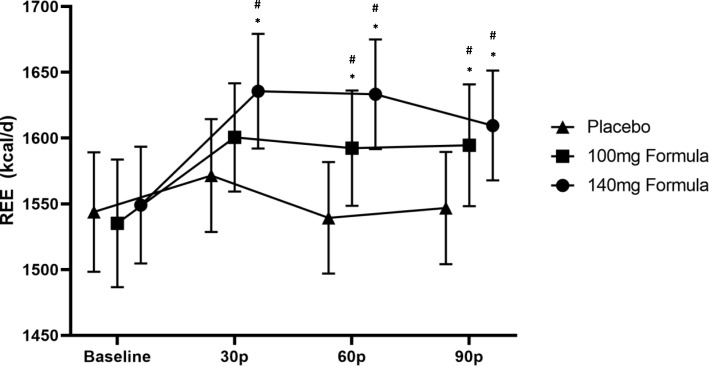
Resting energy expenditure (kcal/day); data (*n* = 32) are expressed as mean ± SE. # different than baseline; * different than placebo (*p* < 0.05). 30p = measurement average at 30 min post drink ingestion; 60p = measurement average at 60 min post drink ingestion; 90p = measurement average at 90 min post drink ingestion

A significant trial × time interaction was found for REE (*p* < 0.01). Follow-up repeated measure ANOVAs revealed differences across trials and time points (Fig. 2). There were no significant differences at baseline across trials between the 140 mg formula, 100 mg formula, and placebo trials (*p* = 0.76). However, REE was significantly higher at 30 min post-ingestion, for the 140 mg formula trial as compared to the 100 mg formula (*p* = 0.02; *d* = 0.490) and placebo (*p* < 0.01; *d* = 0.830) trials, which did not significantly differ between each other (*p* = 0.06). At 60 min post-ingestion, REE values were significantly greater for the 140 mg formula compared to 100 mg formula (*p* = 0.02; *d* = 0.439) and placebo (*p* < 0.01; *d* = 0.925). A significant difference for REE was also found at 60 min post-ingestion between the 100 mg formula and placebo (*p* = 0.01; *d* = 0.508). REE for both of the caffeinated formulas was significantly greater at 90 min post-ingestion compared to placebo (140 mg: *p* < 0.01; *d* = 0.788; 100 mg: *p* = 0.03; *d* = 0.468), but not different between each other (*p* = 0.39). Across time, the 140 mg formula and the 100 mg formula both significantly increased REE at 30 (*p* = 0.01, *d* = − 0.756; *p* = 0.03, *d* = 0.546, respectively), 60 (*p* = 0.01, *d* = − 0.749; *p* = 0.03, *d* = − 0.518, respectively), and 90 min post-ingestion (*p* = 0.02, *d* = − 0.524; *p* = 0.03, *d* = − 0.526) compared to baseline. No significant differences over time were shown for REE during the placebo trial (*p* = 0.11).

Total REE estimated from area under the curve analysis demonstrated significant differences between all trials (*p* < 0.01; 140 mg = 101.0 ± 14.7 kcal; 100 mg = 99.1 ± 15.2 kcal; placebo = 97.3 ± 15.2 kcal). Post hoc revealed that 140 mg formula demonstrated the greatest caloric expenditure compared to 100 mg formula (*p* = 0.02; *d* = 0.462) and placebo (*p* = < 0.01; *d* = 0.911) and that 100 mg formula was significantly greater than placebo (*p* = 0.02; *d* = 0.449).

**Fig. 3:**
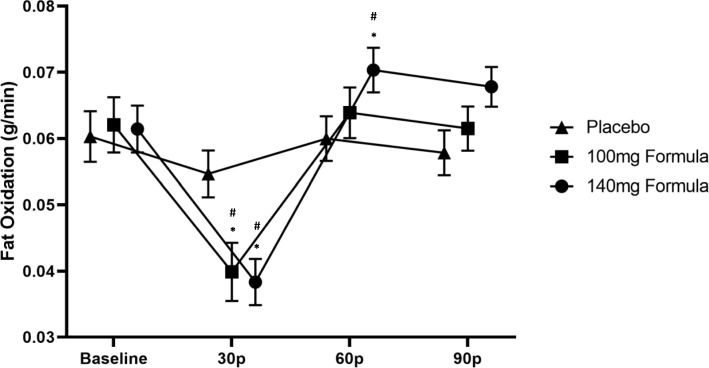
Resting fat oxidation (g/min); data (*n* = 32) are expressed as mean ± SE. # different than baseline; * different than placebo (*p* < 0.05). 30p = measurement average at 30 min post drink ingestion; 60p = measurement average at 60 min post drink ingestion; 90p = measurement average at 90 min post drink ingestion

A significant trial × time interaction was found for RFO (*p* < 0.01; Fig. 3). Follow-up repeated measure ANOVAs revealed differences across trials and time points. At baseline, no significant differences were noted between the 140 mg formula, 100 mg formula, and placebo trials (*p* = 0.92). However, at 30 min post-ingestion, a significantly lower RFO was noted in the 100 mg (*p* < 0.01, *d* = − 0.702) and 140 mg (*p* < 0.01; *d* = − 0.841) formulas compared to placebo, while no significant differences were noted between the 140 mg and 100 mg formulas (*p* = 0.56; *d* = − 0.104). At 60 min post-ingestion, RFO values changed and were significantly higher for the 140 mg formula compared to placebo (*p* = 0.02; *d* = 0.504) but were not different from the 100 mg formula (*p* = 0.28*; d* = 0.269). Moreover, 100 mg was not significantly different than placebo (*p* = 0.28*; d* = 0.199). Results were similar at 90 min post-ingestion with RFO being significantly higher for the 140 mg formula compared to placebo (*p* = 0.03; *d* = 0.486), while no differences were shown between the 100 mg formula and the 140 mg formula (*p* = 0.16*; d* = 0.321) or 100 mg formula and placebo (*p* = 0.22*; d* = 0.220). Compared to baseline, RFO decreased significantly at 30 min post-ingestion during the 140 mg formula trial (*p* < 0.01; *d* = 1.030) and increased significantly at 60 min post-ingestion (*p* = 0.03*; d* = − 0.485). No significant difference was noted between baseline and 90 min post-ingestion (*p* = 0.08*; d* = − 0.377). For the 140 mg formula, RFO values at 30 min were significantly lower than 60 (*p* < 0.01; *d* = − 2.159) and 90 min post-ingestion (*p* < 0.01; *d* = − 2.118). The 100 mg formula displayed similar results with significantly lower RFO at 30 min post-ingestion compared to baseline (*p* < 0.01; *d* = 1.042); however, no significant differences were shown at 60 (*p* = 1.00; *d* = − 0.088) and 90 min post-ingestion (*p* = 1.00; *d* = 0.025) compared to baseline. Significantly higher RFO values were shown at 60 (*p* < 0.01; *d* = − 1.166) and 90 min (*p* < 0.01; *d* = − 1.274) compared to 30 min post-ingestion for the 100 mg formula, while values at 60 were not significantly different than 90 min post-ingestion (*p* = 0.64; *d* = 0.225). Placebo did not significantly change across time (*p* = 0.11).

### Blood venous sampling and glycerol analysis

**Fig. 4:**
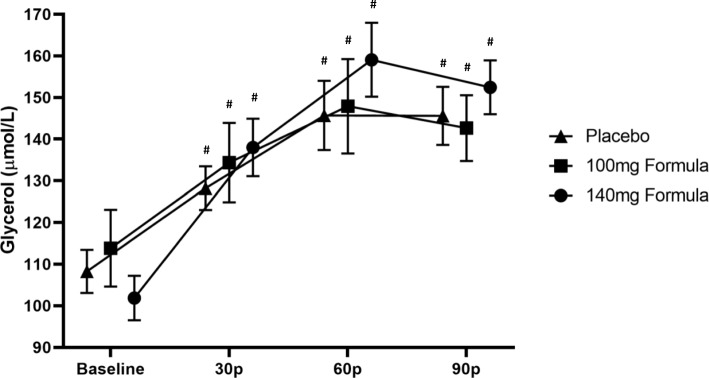
Resting serum glycerol concentration (μmol/L); data (*n* = 30) are expressed as mean ± SE. # different than baseline (*p* < 0.05). 30p = measurement taken at 30 min post drink ingestion; 60p = measurement taken at 60 min post drink ingestion; 90p = measurement taken 90 min post drink ingestion

No significant interaction (trial x time) was observed for serum glycerol concentration (*p* = 0.09; Fig. 4). However, a significant main effect of time was observed (*p* < 0.01). Post hoc revealed that serum glycerol was significantly elevated at 30 (*p* < 0.01, *d* = − 1.000), 60 (*p* < 0.01, *d* = − 1.257), and 90 min post-ingestion (*p* < 0.01, *d* = − 1.407) relative to baseline. Additionally, glycerol concentrations were significantly increased at 60 (*p* = 0.01, *d* = − 0.568) and 90 min (*p* = 0.01, *d* = − 0.574) relative to 30 min post-ingestion. No significant differences were observed between 60 and 90 min post-ingestion (*p* = 0.56).

### Graded exercise test, indirect calorimetry, and Fat_max_ calculation

**Fig. 5:**
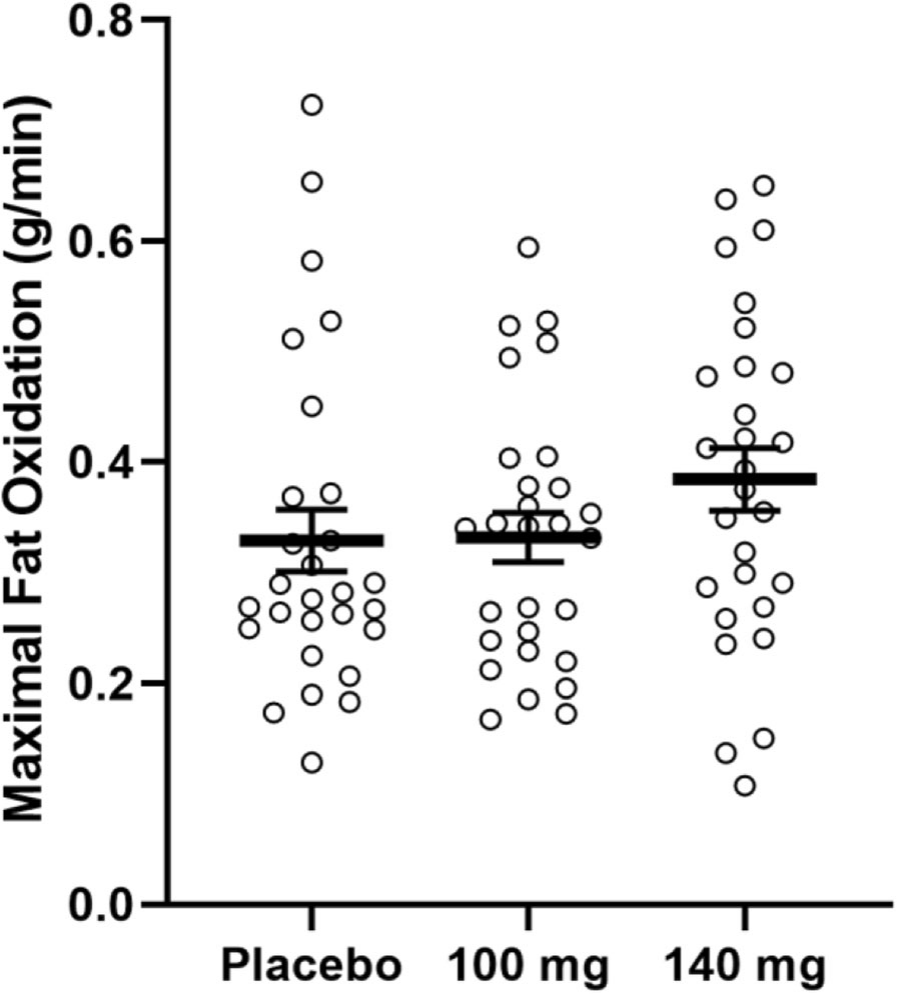
Maximal fat oxidation during exercise (g/min); data (*n* = 28) are expressed as mean ± SE

There were no significant differences for V̇O_2max_ (*p* = 0.12), Fat_max_ (*p* = 0.22), MFO across trials (*p* = 0.05; Fig. 5), and EE across drinks (*p* = 0.08; Table 2).

**Table 2:** GXT variables

Variable	Placebo	100 mg Formula	140 mg Formula
V̇O_2max_ (mL/kg·min)	36.5 ± 1.3	37.5 ± 1.3	38.2 ± 1.5
Fat_max_ (W)	60.3 ± 3.4	64.5 ± 3.3	65.6 ± 3.7
MFO (g/min)	0.33 ± 0.03	0.33 ± 0.2	0.38 ± 0.03
EE (kcal)	168.7 ± 9.2	172.9 ± 9.4	182.7 ± 10.3

Data is expressed as mean ± SE. MFO = maximal fat oxidation; EE = total energy expenditure during exercise

## Discussion

The main finding of this study was that a decrease from 140 mg to 100 mg of caffeine within the examined thermogenic fitness drink (TFD) formulas appear to promote changes in energy expenditure (REE) and fat metabolism (RFO) at rest in healthy individuals. Results showed a significant increase in total REE over the 90-min trial for the 140 mg formula, as compared to the 100 mg formula (+ 2%) and placebo (+ 4%). Our results are similar to the findings of Mendel and Hofheins [33], who compared the metabolic effects of a similar TFD (200 mg of caffeine) to a caffeine containing diet soft drink (45 mg) 3-h post-ingestion. Their results indicated a significant increase in resting V̇O_2_ in L/min for the TFD at all time points, as compared to no significant change when consuming the diet soft drink [33]. In the present study, our formulas displayed a 5.8% increase for 140 mg formula and a 3.9% increase for the 100 mg formula at 60 min post-ingestion; whereas Mendel and Hofheins [33] reported an increase of 13.8% for the 200 mg TFD at the same time point.

Conversely, Gonzalez et al. [34] administered regular and time release caffeine containing supplements with 194 mg of caffeine and did not find any significant difference for REE or glycerol over the course of 8 h; however, it must be noted that participants were habitual caffeine consumers with a reported daily average intake greater than 200 mg. In agreement with this study [34] and Dalbo et al. [15], we also found no significant differences for changes in glycerol between TFDs and placebo, although a significant increase over time was displayed possibly due to fat cycling provoked by the fasted conditions (10 kcal for caffeinated formulas and 0 kcal for placebo) [35]. Alternatively, both Graham et al. [36] and Astrup et al. [16] showed a significantly greater serum concentration of glycerol during rest following the ingestion of higher caffeine doses (6 mg/kg and 200 mg of caffeine, respectively). However, these comparisons were made with placebo formulas containing dextrose and lactose, respectively, which could have possibly increased blood glucose and insulin concentrations and consequently reduced lipolysis for the placebo trials. Provided that participants in this study ingested a much lower relative amount of caffeine per body mass (1.5 ± 0.3 mg/kg for 100 mg formula and 2.1 ± 0.4 mg/kg for 140 mg formula) and that some evidence suggests that serum glycerol rise is not always a sensitive measure of lipid mobilization, we also estimated RFO by the use of indirect calorimetry and stoichiometric equations [14].

Interestingly, results from these estimates presented oscillatory effect for substrate utilization throughout the 90 min of rest. Initially, at 30 min after ingestion, RFO decreased for both caffeinated formulas suggesting greater carbohydrate utilization during this time period. A similar response has been reported for a different noncaloric energy drink formula containing 114 mg of caffeine, taurine, vitamins and, aspartame and acesulfame K with the hypothesis given that the artificial sweeteners typically present in most of these formulas may have an impact on the respiratory quotient via sensorial stimulation and possibly insulin secretion [37]. Nonetheless, the present study found that RFO was significantly increased for the 140 mg formula, but not for 100 mg formula at 60 min post-ingestion. This finding suggests that a dosage between 100 mg and 140 mg of caffeine may represent a minimum threshold for the tested formula to impact fat metabolism at rest for the participants in this study.

The effects of caffeine on energy expenditure (EE) and the maximal fat oxidation (MFO) during exercise were recently studied by Gutiérrez-Hellín and Del Coso [18] who reported an increase in MFO rates (between 30 and 70% V̇O_2max_) following consumption of 3 mg/kg of p-synephrine and caffeine, but no difference for Fat_max_ (the power output reached at MFO) or EE. As a stimulant of the central nervous system, caffeine was shown to influence metabolism, which may have affected beta-adrenoreceptors and fat oxidation during exercise. Nonetheless, no significant differences were reported for EE, Fat_max_, and MFO in this study. Thus, higher amounts of caffeine or a shorter amount of time between ingestion and exercise (< 2 h) may be required to substantially affect MFO during incremental exercise.

Most notably, this is the first study to our knowledge to show significant differences in REE and RFO between absolute amounts of 100 mg and 140 mg of caffeine as part of a TFD formula in physically active men and women and to have a repeated measures design to do so. Our results suggest a minimum threshold that is likely to be above 100 mg of caffeine and that a small decrement in the caffeine content of a commercially available TFD may influence thermogenesis and fat metabolism at rest in the current cohort of participants. It is important to note that weight loss and/or fat loss cannot be directly inferred from the current results, and it is unknown whether increased REE at rest in a chronic energy deficit would ultimately result in fat loss. Therefore, we propose that future work evaluate the acute and chronic differences of different formulas with varying doses of caffeine on energy expenditure and fat metabolism at rest and during exercise.

## Conclusions

Acute ingestion of a TFD containing either 140 mg or 100 mg of caffeine significantly increased resting metabolic rate in the current cohort of participants, while the TFD containing 140 mg of caffeine appears to have also increased resting fat oxidation. However, no significant differences were shown for GXT variables suggesting that higher dosages of caffeine or a shorter duration between ingestion and subsequent physical activity may be required to influence energy expenditure and fat metabolism during exercise.

## Data Availability

The datasets used and/or analysed during the current study are available from the corresponding author on reasonable request.
